# Glasgow prognostic score is superior to other inflammation-based scores in predicting survival of diffuse large B-cell lymphoma

**DOI:** 10.18632/oncotarget.20832

**Published:** 2017-09-11

**Authors:** Xiaoxiao Hao, Yongqiang Wei, Xiaolei Wei, Lizhi Zhou, Qi Wei, Yuankun Zhang, Weimin Huang, Ru Feng

**Affiliations:** ^1^ Department of Hematology, Nanfang Hospital, Southern Medical University, Guangzhou, China; ^2^ Department of Biostatistics, School of Public Health, Southern Medical University, Guangzhou, China

**Keywords:** diffuse large B-cell lymphoma, glasgow prognostic score, inflammation-based prognostic score

## Abstract

Inflammation-based prognostic scores, such as the glasgow prognostic score (GPS), prognostic index (PI), prognostic nutritional index (PNI), neutrophil lymphocyte ratio (NLR) and platelet lymphocyte ratio (PLR) were related to survival in many solid tumors. Recent study showed that GPS can be used to predict outcome in diffuse large B-cell lymphoma (DLBCL). However, other inflammation related scores had not been reported and it also remained unknown which of them was the most useful to evaluate the survival in DLBCLs. In this retrospective study, a number of 252 newly diagnosed and histologically proven DLBCLs from January 2003 to December 2014 were included. The high GPS, high PI, high NLR, high PLR and low PNI were all associated with poor overall survival (*p* < 0.05) and event-free survival (*p* < 0.05) in univariate analysis. Multivariate analysis indicated that GPS (HR = 1.781, 95% CI = 1.065–2.979, *p* = 0.028) remained an independent prognostic predictor in DLBCL. The c-index of GPS (0.735, 95% CI = 0.645–0.824) was greater than that of PI (0.710, 95% CI = 0.621–0.799, *p* = 0.602), PNI (0.600, 95% CI = 0.517–0.683, *p* = 0.001), PLR (0.599, 95% CI = 0.510–0.689, *p* = 0.029) and NLR (0.572, 95% CI = 0.503–0.642, *p* = 0.005) by Harrell's concordance index. Especially in DLBCLs treated with R-CHOP, GPS still remained the most powerful prognostic score when comparing with others (*p* = 0.001 and *p* < 0.001, respectively for OS and EFS). In conclusion, it is indicated that inflammation-based prognostic scores such as GPS, PI, NLR, PNI and PLR all could be used to predict the outcome of DLBCLs. Among them, GPS is the most powerful indicator in predicting survival in DLBCLs, even in the rituximab era.

## INTRODUCTION

Diffuse large B-cell lymphoma (DLBCL) is one of the most common subtypes non-Hodgkin lymphomas, characterized by heterogeneity in clinical, immunophenotypic, clinical response and pathogenetics [1]. The combination of rituximab with cyclophosphamide, doxorubcin, vincristine and prednisone (R-CHOP) have greatly improved the outcome in DLBCL [2, 3]. Nevertheless, still a number of patients with DLBCL undergo relapse or refractory to the standard first-line treatment, which highlights that it is important to construct a prognostic model to identify those patients, helps to guide the choice of initial treatment and allows for appropriate stratification and interpretation of clinical trials.

Recent studies have begun to unravel the mechanism linking the host inflammatory response to tumor growth, invasion and metastasis [4]. Markers of host response to inflammatory, such as C-reactive protein (CRP), albumin (ALB), neutrophil, lymphocyte and platelet were reported to correlate with survival in many tumors [5, 6]. Meanwhile, increasing evidence indicated that inflammation-based scores including GPS (a combination of the serum CRP and ALB), PLR (platelet lymphocyte ratio), NLR (neutrophil lymphocyte ratio), PI (a mix of CRP and white blood cell) and PNI (Onodera's Prognostic Nutritional Index) were useful for predicting outcome in various malignancies [7–10].

A well-known inflammatory cytokine, interleukin-6 (IL-6) has been implicated in a wide variety of human biological function such as B-cell differentiation, hematopoiesis and platelet production as well as acute and chronic inflammation related diseases and its related signaling pathways have been identified to contribute to tumor growth, invasion and metastasis [11, 12]. IL-6 modulates the synthesis of positive (such as CRP) and negative (like serum ALB) acute phase reactants [13]. It is reported that IL-6 level was associated with poor overall survival in DLBCLs [14]. And blockade of inflammation cytokines greatly inhibits the progression of various tumors [15]. GPS has been used to predict outcome in DLBCL [7]. However, other inflammation-based scores including NLR, PLR, PI and PNI have not been explored in DLBCL. These cost-effective biomarkers are used routinely in the clinical setting and might be used to provide additional information for patients’ outcome. Therefore, we conducted this retrospective study to explore the prognostic value of GPS, NLR, PLR, PI and PNI in DLBCL patients to identify which one of them is the most useful for evaluating the outcome of DLBCL.

## RESULTS

### Basic clinical characteristics

In the all 252 included patients, there were 165 men and the ratio of male to female was 1.89, with a median age at the time of diagnosis was 49-year-old (rang from 16 y to 82 y). Fifty-eight patients (23.0%) were older than 60 years old and 97 (38.4%) were presented with B symptoms at the time of diagnosis. One-hundred twenty-nine (51.2%) patients had elevated LDH and 155 (61.5%) were in advanced clinical stage. Based on the International Prognostic Index (IPI), 162 (64.3%) and 90 (35.7%) were subtyped into low/low-intermediate and high/high-intermediate risk groups, respectively. The immunohistochemistry (IHC) indicated that 117 (46.4%) were GCB, while others were non-GCB according to Hans^’^ classification. Based on treatment grouping, there were 150 patients treated with R-CHOP, while others received CHOP regimen.

### The prevalence and prognostic value of Inflammation-based prognostic scores in DLBCL

With regard to PLR, 111 (44.0%) patients, 104 (41.3%) patients and 37 (14.7%) patients were grouped to PLR 0, PLR 1 and PLR 2, respectively. According to NLR classification, 64 (25.4%) patients were NLR 1. One-hundred twenty-seven (50.4%) and 125 (49.6%) were grouped into PNI 0 and PNI 1, respectively. The PI grouped a total of 88 (43.3%), 69 (34.0%) and 46 (22.7%) patients to PI 0, PI 1 and PI 2, respectively. According to GPS subgroup, 83 (40.9%) patients, 70 patients (34.5%) and 50 patients (24.6%) were grouped to GPS 0, GPS 1 and GPS 2, respectively. (Data showed in Table 2).

**Table 2:** Revalence of inflammation-based prognostic scores

Types of Inflammation-based prognostic score	Scores	NO. (%)
Glasgow prognostic score (GPS)		
CRP ≤ 10 mg/L and ALB ≥ 35 g/l	0	83 (40.9%)
CRP or ALB only one abnormal	1	70 (34.5%)
CRP > 10 mg/l and ALB < 35g/l	2	50 (24.6%)
Prognostic Index (PI)		
CRP ≤ 10 mg/l and WBC ≤ 11*10^9/l	0	88 (43.3%)
CRP or WBC only one abnormal	1	69 (34.0%)
CRP > 10 mg/l and WBC > 11*10^9/l	2	46 (22.7%)
Prognostic Nutritional Index (PNI)		
ALB+5*total lymphocytes ≥ 45	0	127 (50.4%)
ALB+5*total lymphocytes < 45	1	125 (49.6%)
Neutrophil Lymphocyte Ratio (NLR)		
Neutrophil: Lymphocyte < 5:1	0	188 (74.6%)
Neutrophil: Lymphocyte ≥ 5:1	1	64 (25.4%)
Platelet Lymphocyte Ratio (PLR)		
Platelet:Lymphocyte < 150:1	0	111 (44.0%)
Platelet:Lymphocyte = 150–300:1	1	104 (41.3%)
Platelet:Lymphocyte > 300:1	2	37 (14.7%)

**Figure 1:**
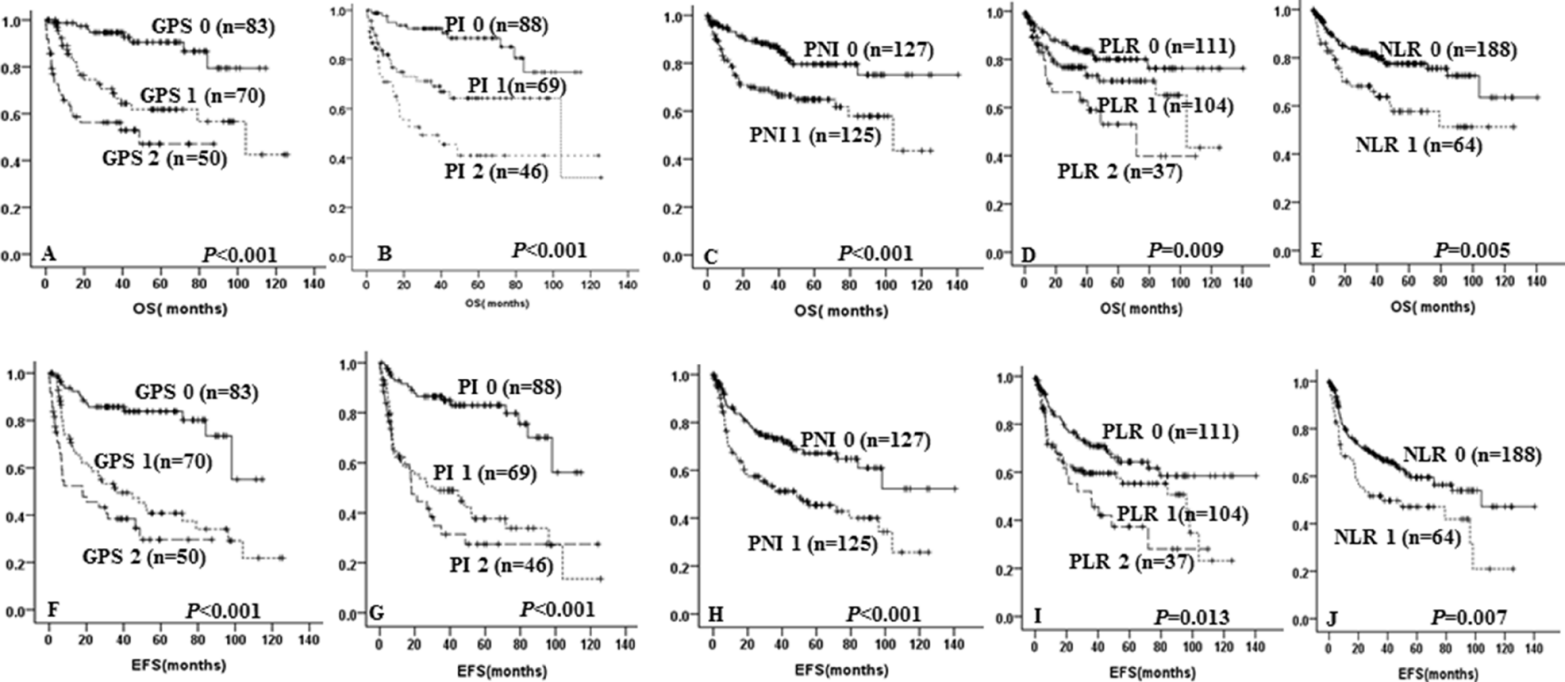
Kaplan-Meier survival curve of overall survival and event-free survival in DLBCL patients according to GPS (**A** and **F**), PI (**B** and **G**), PNI (**C** and **H**), PLR (**D** and **I**), NLR (**E** and **J**).

Univariate analysis revealed that high GPS (*p* < 0.001 and *p* < 0.001, Figure 1A and 1F), high PI (*p* < 0.001 and *p* < 0.001, Figure 1B and 1G), high NLR (*p* = 0.005 and *p* = 0.007, Figure 1E and 1J), high PLR (*p* = 0.009 and *p* = 0.013, Figure 1D and 1I) and low PNI (*p* < 0.001 and *p* < 0.001, Figure 1C and 1H) were all significantly associated with both inferior OS and EFS, respectively. Multivariate analysis showed that high GPS independent of other inflammation-based prognostic scores and IPI was an unfavorable predictor of OS (HR = 1.781, 95% CI = 1.065–2.979, *p* = 0.028) and EFS (HR = 1.763, 95% CI = 1.165–2.667, *p* = 0.007). (Data showed in Table 4).

**Table 4:** Multivariate Cox regression analysis for survival of patients with DLBCL

Prognostic factors	HR	95% CI	*P*-value
Overall survival			
IPI 3–5	1.726	0.9991–3.007	0.054
GPS	1.781	1.065–2.979	0.028
NLR	0.935	0.491–1.783	0.839
PLR	1.027	0.664–1.589	0.905
PI	1.378	0.834–2.272	0.208
PNI	1.697	0.834–3.455	0.144
Event-free survival			
IPI 3–5	1.359	0.865–2.133	0.183
GPS	1.763	1.165–2.667	0.007
NLR	0.967	0.575–1.627	0.899
PLR	1.056	0.747–1.492	0.758
PI	1.368	0.915–2.045	0.127
PNI	1.229	0.710–2.126	0.461

HR hazard ratio, 95% CI 95% Confidence Interval.

To assess the discriminatory ability of GPS, NLR, PLR, PNI and PI in predicting survival, the Harrell's concordance index was constructed. There was a greatly better performance for GPS (0.735, 95% CI = 0.645–0.824) than PI (0.710, 95% CI = 0.621–0.799, *p* = 0.602), PNI (0.600, 95% CI = 0.517–0.683, *p* = 0.001), PLR (0.599, 95% CI = 0.510–0.689, *p* = 0.029) and NLR (0.572, 95%CI = 0.503–0.642, *p* = 0.005) in the prognostic model for OS and EFS (GPS: 0.697, 95% CI = 0.628–0.765) (Data showed in Table 3).

**Table 3:** Predictive scores regarding survival investigated by Harrell's concordance index (C-index)

Inflammation-based prognostic scores	C-index	95% CI	*P*-value*
Overall-survival			
GPS	0.735	0.645–0.824	
PI	0.710	0.621–0.799	0.602
PNI	0.600	0.517–0.683	0.001
PLR	0.599	0.510–0.689	0.029
NLR	0.572	0.503–0.642	0.005
Event-free survival			
GPS	0.697	0.628–0.765	
PI	0.692	0.624–0.760	0.871
PNI	0.652	0.499–0.625	< 0.001
PLR	0.570	0.510–0.639	0.006
NLR	0.546	0.493–0.600	0.001

C-index Harrell's concordance index, 95% CI 95% Confidence Interval, *compared to GPS.

### GPS was the most useful predictor for survival in DLBCL among these inflammation-based prognostic scores

Our results indicated that high GPS was significantly related with LDH (*p* < 0.001) level, B symptoms (*p* < 0.001), ferritin (*p* = 0.010), WBC (*p* < 0.001), low Hb level (*p* < 0.001), advanced clinical stage (*p* < 0.001), higher IPI (3–5) (*p* = 0.015) compared with those with lower GPS. While the other clinical characteristics including gender, performance status, cell of origin, PLT and treatment showed no significant differences in DLBCLs among GPS groups (*p* > 0.05). (Data showed in Table 1).

**Table 1:** Clinical characteristics of patients according to GPS score

Characteristics	No. (%)	GPS 0 (*n*%)	GPS 1 (*n*%)	GPS 2 (*n*%)	*P*-Value
Age					0.113
≤ 60 y	159 (78.3%)	69 (83.1%)	56 (80.0%)	34 (68.0%)	
> 60 y	44 (21.7%)	14 (16.9%)	14 (20.0%)	18 (32.0%)	
Gender					0.415
Female	68 (33.5%)	32 (38.6%)	22 (31.4%)	14 (28.0%)	
Male	135 (66.5%)	51 (61.4%)	48 (68.6%)	36 (72.0%)	
Performance Status					0.552
0–1	150 (73.9%)	63 (75.9%)	53 (76.7%)	34 (68.0%)	
≥ 2	53 (26.1%)	20 (24.1%)	17 (23.3%)	16 (32.0%)	
LDH					< 0.001
Normal	98 (47.8%)	56 (67.5%)	30 (42.9%)	12 (24.0%)	
Elevated	105 (52.2%)	27 (32.5%)	40 (57.1%)	38 (76.0%)	
Ann Arbor Status					< 0.001
I/II	80 (39.4%)	48 (57.8%)	20 (28.6%)	12 (24.0%)	
III/IV	123 (60.5%)	35 (42.2%)	50 (71.4%)	38 (76.0%)	
IPI					0.015
0–2	128 (63.1%)	58 (69.9%)	47 (67.5%)	23 (46.0%)	
3–5	75 (36.9%)	25 (30.1%)	23 (32.9%)	27 (54.0%)	
B symptoms					< 0.001
No	124 (61.1%)	68 (81.9%)	42 (60.0%)	14 (28.0%)	
Yes	79 (38.9%)	15 (18.1%)	28 (40.0%)	36 (72.0%)	
Extranodal Sites					0.071
0–1	114 (56.2%)	41 (49.4%)	47 (67.1%)	26 (52.0%)	
≥ 2	89 (43.8%)	42 (50.6%)	23 (32.9%)	24 (48.0%)	
COO					0.390
GCB	89 (43.8%)	40 (48.2%)	31 (44.3%)	18 (36.0%)	
non-GCB	114 (56.2%)	43 (51.8%)	39 (55.7%)	34 (64.0%)	
ALB					< 0.001
≥ 35g/L	129 (63.5%)	73 (88.0%)	50 (71.4%)	6 (12.0%)	
< 35g/L	74 (36.5%)	10 (12.0%)	20 (28.6%)	44 (88.0%)	
CRP					< 0.001
Normal	88 (43.3%)	78 (94.0%)	8 (11.4%)	2 (4.0%)	
Elevated	115 (56.7%)	5 (6.0%)	62 (88.6%)	48 (96.0%)	
Ferritin					0.010
Normal	81 (47.6%)	46 (60.5%)	22 (38.6%)	13 (35.1%)	
Elevated	89 (52.4%)	30 (39.5%)	35 (61.4%)	24 (64.9%)	
WBC					< 0.001
Normal	134 (66.0%)	62 (74.7%)	51 (72.9%)	21 (42.0%)	
Elevated	69 (34.0%)	21 (25.3%)	19 (27.1%)	29 (58.0%)	
PLT					0.175
Normal	150 (73.9%)	63 (75.9%)	55 (73.6%)	32 (64.0%)	
Elevated	53 (26.1%)	20 (24.1%)	15 (21.4%)	18 (36.0%)	
Hb					< 0.001
Normal	168 (82.8%)	79 (95.2%)	59 (84.3%)	30 (60.0%)	
Elevated	35 (17.2%)	4 (4.8%)	11 (15.7%)	20 (40.0%)	
Treatment					0.454
CHOP	80 (39.4%)	28 (33.7%)	29 (41.4%)	23 (46.0%)	
R-CHOP	123 (60.6%)	55 (66.3%)	41 (58.6%)	27 (54.0%)	

GPS glasglow prognostic score, PNI Prognostic Nutritional Index, PI Prognostic Index, NLR Neutrophil Lymphocyte Ratio, PLR Platelet Lymphocyte Ratio, LDH lactate dehydrogenase, IPI International Prognostic Index, CRP C-reactive protein, GCB germinal center B-cell-like, non-GCB non-germinal center B-cell-like, ALB Albumin, WBC white blood cell counts, PLT platelet count, Hb Hemoglobin, CHOP (cyclophosphamide doxorubicin vincristine and prednisone), R-CHOP (rituximab plus cyclophosphamide doxorubicin vincristine and prednisone), COO cell of origin, IPI international prognostic index.

It is indicated that patients with GPS 1 and GPS 2 were significantly with unfavorable 5-year OS (63.2% ± 6.7% vs 46.0% ± 8.8% vs 90.6% ± 3.8%, *p* < 0.001, Figure 1A) and 5-year EFS (48.4 ± 6.6% vs 30.4% ± 8.1% vs 83.8% ± 4.3%, *p* < 0.001, Figure 1F) when compared with GPS 0.

**Figure 2:**
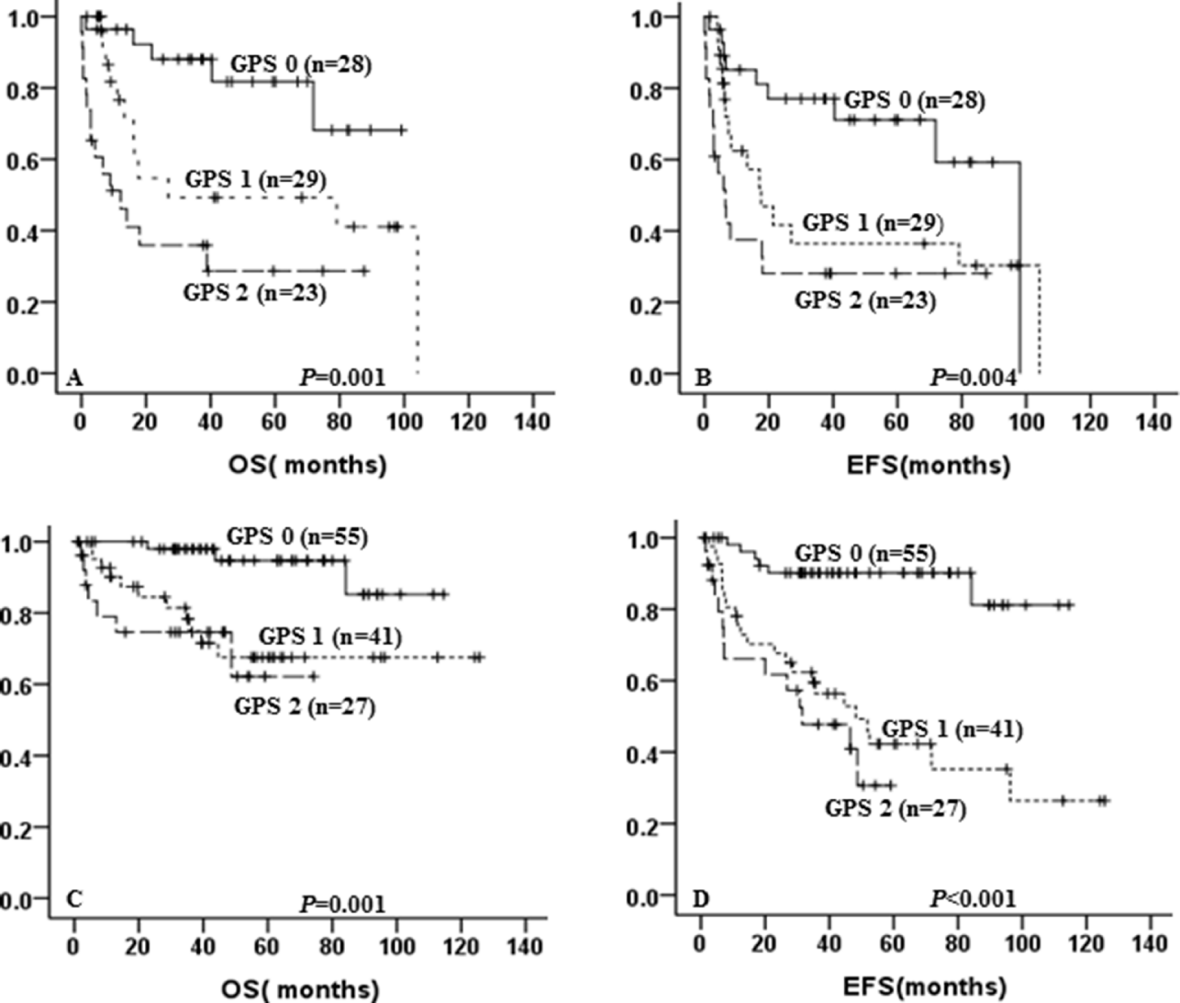
Kaplan-Meier curve for overall survival (OS) and event-free survival (EFS) according to GPS and treatment OS (**A**) and EFS (**B**) according to GPS of DLBCL patients treated with CHOP; OS (**C**) and EFS (**D**) according to GPS of DLBCL patients treated with R-CHOP.

Allocated to GPS, there were 123 patients treated with R-CHOP, while others received CHOP. In patients treated with CHOP, GPS 1–2 showed a tendency to predict poor for 5-year OS (49.3% ± 11.5% vs 28.7% ± 10.65% vs 81.8% ± 8.6%, *p* < 0.001, Figure 2A) and 5-year EFS (36.4% ± 10.7% vs 28.1% ± 9.6% vs 71.1% ± 9.5%, *p* = 0.004, Figure 2B) as compared with GPS 0. In the R-CHOP group, elevated GPS was still associated with shorter 5-year OS (67.5% ± 8.8% vs 62.2% ± 13.6% vs 94.7% ± 3.8%, *p* = 0.001, Figure 2C) and 5-year EFS (42.3% ± 8.6% vs 30.7% ± 12.1% vs 90.2% ± 4.2%, *p* < 0.001, Figure 2D).

All patients were divided by low/low intermediate group and high intermediate/high group according to IPI score. In the high intermediate/high group, patients with higher GPS score implied a poor of OS and EFS (*p* = 0.001, *p* = 0.001, respectively for OS and EFS). In the low/low intermediate group, patients with higher GPS was still associated with unfavorable OS and EFS (*p* < 0.001, *p* < 0.001, respectively for OS and EFS).

## DISCUSSION

In the present study, we evaluated the predictability of inflammation-based scores including GPS, NLR, PLR, PI and PNI in de novo DLBCL patients, explored evidence for those with system inflammatory patients who might own poor outcome and aimed to find individualized treatment. In this retrospective study, it is suggested that high GPS, PI, NLR, PLR and low PNI were all associated with shorter OS and EFS in DLBCLs in univariate model. In multivariate analysis, GPS independent of IPI and other inflammation-based scores was a powerful predictor. In terms of Harrell's concordance index, GPS also showed superiority when discriminating good from poor as compared with other scores in DLBCL patients. The robust prognostic value of GPS retained in DLBCL patients treated with either R-CHOP or CHOP.

It is now accepted that the tumor microenvironment is mostly orchestrated by inflammatory cells like macrophage, neutrophil, monocyte, lymphocyte and other cells, which played an vital role in the tumor process, proliferation, progression and metastasis [10, 16, 17]. Previous studies have proved that NLR is an independent predictor in gastric tumor [18]. However, it is still controversial, Deshen et al demonstrated that NLR was not correlated with OS and EFS in post-operated gastric cancer patients [19]. In our retrospective study, we also demonstrated that NLR was not an independent predictor in DLBCLs. Due to the differences among studies including different cutoff values, the types, the stages of disease, pre-or postoperative patients as well as combination of chemical-therapy, the prognostic value of NLR has been controversial [8, 20, 21]. It may be meaningful to explore large-scale studies to validate whether NLR be an independent predictor.

PLR has been showed to be an significant predictor in colon cancer and ovarian cancer [22, 23]. Whereas, studies from Deshen et al suggested that PLR was not an independent prognostic factor in gastric cancer patients, which we also confirmed in the present study [19]. However, it might be worthy to investigate larger-scale study to validate whether PLR was an independent index.

Although, studies indicated that PI and PNI have an prognostic value in lung cancer and prostate cancer [9, 24]. The predictive value of PNI and PI on survival of DLBCLs have not been fully investigated. Our study revealed that low PNI was related with shorter OS and EFS, but multivariate analysis proved it was not an independent predictor and C-index of PNI was less than that of GPS when assessed by Harrell's concordance index (0.600 vs 0.735, respectively). We also found that increased PI was associated with poor OS and EFS, but multiple analysis revealed that PI was not an independent indicator.

GPS, a combination of CRP and ALB, is an excellent index of system inflammation and malnutrition. Previous studies have proved that GPS as a solid prognostic factor in various tumors like Cervial cancer, non-small cell lung cancer, gallbladder cancer and advanced biliary tract cancer [25–28]. However, the study of GPS in lymphoma was relatively rare. In the present study, we found GPS was an independent predictor of survival in DLBCL patients, which was consistent with the results of previous studies evaluating the prognostic of the GPS in extranodal natural killer (NK)/T-cell lymphoma (ENKL) and DLBCLs [7, 29]. Moreover, Harrell's concordance index highlighted that GPS was superior to others in terms of discriminating good from poor. These results were kept in line with the work of Akihiko Kato, Proctor and Pan Q [24, 30, 31].

However, there are a few limitations should be acknowledged in our study. There are some potential confounding factors that can't be avoided in this retrospective study. To minimize the inherent biases of the retrospective study, we included only patients with de novo DLBCL treated with the first-line chemotherapy and excluded patients suffering detectable acute inflammation, central nerve system DLBCL, PMBCL, intravascular and testicular lymphoma.

In conclusion, this retrospective study revealed that GPS, PNI, PI, NLR and PLR all have prognostic value in DLBCL. GPS was superior to the other inflammation-based scores (PNI, PI, NLR and PLR) in predicting survival of DLBCLs. Especially in R-CHOP group, GPS still showed a robust predictor. The results of our study and the existing validation literatures might suggest that the cost-efficiency predictor GPS should be assessed at the time of diagnosis in DLBCL.

## MATERIALS AND METHODS

### Patients

In our retrospective study, we reviewed the medical record of DLBCL patients from January 2003 to December 2014 at Nanfang Hospital affiliated to Southern Medical University. From the observed 366 patients, 252 were included according to the including and excluding criteria. All the patients’ blood count and ALB were available. CRP was available in 203 patients. All cases were pathologically diagnosed according to the World Health Organization classification criteria and treated with R-CHOP, CHOP or CHOP-like. Cases were excluded if primary central nervous system DLBCL, primary mediastinal large B-cell lymphoma (PMBCL), transformed DLBCL or acquired immunodeficiency. Patients suffering detectable acute inflammation or chronic active inflammatory disease were also excluded. All laboratory data were obtained at diagnosis. The inflammation-based prognostic scores GPS, NLR, PLR, PI and PNI were showed in Table 2. The Ethics Committee of Nanfang hospital approved the study and all patients had provided written informed consent themselves or their guardians prior to treatment allowing the use of their medical records for medical research.

### Statistics analysis

The Mann-Whitney *U* test, Chi-square and Kruskal-Wallis H test were used for assessment of differences among groups. Overall survival (OS) was defined as the time of diagnosis until death as a result of any cause or the date last known to be alive. Event-free survival (EFS) was calculated from the date of diagnosis to the documented disease relapse, progression or death or last follow-up time. Survival curves were generated by Kaplan-Meier method with the log-rank test for comparison of differences. Covariates like IPI and inflammation-based prognostic scores GPS, NLR, PLR, PI and PNI were included in the multivariate analysis. Harrell's concordance index, an extension of the area under the receiver operative curve (AUC) to survival data, was used to assess the predictive accuracy of different biomarkers [12, 13]. *P* ≤ 0.05(two-sided) were considered to be significant. All statistics analysis were carried out by the Statistical Package for Social Sciences (SPSS) for windows 19.0 and R software packages, version 3.1.2.

## Abbreviations

DLBCL: diffuse large B-cell lymphoma
GPS: glasgow prognostic score
PI: prognostic index
PNI: Opodrea prognostic nutritional index
NLR: neutrophil lymphocyte ratio
PLR: platelet lymphocyte ratio
PMBCL: primary mediastinal large B-cell lymphoma
AUC: area under the receiver operative curve
IHC: immunohistochemistry
BM: bone marrow
OS: overall survival
EFS: event-free survival
IPI: International Prognostic Index
CRP: C-reactive protein
GCB: germinal center B-cell-like
non-GCB: non-germinal center B-cell-like
ALB: Albumin
WBC: white blood cell counts
PLT: platelet count
Hb: Hemoglobin
CHOP: cyclophosphamide doxorubicin vincristine and prednisone
R-CHOP: rituximab plus cyclophosphamide doxorubicin vincristine and prednisone
COO: cell of origin
